# Editorial: Computer-aided drug design: Drug discovery, computational modelling, and artificial intelligence

**DOI:** 10.3389/fchem.2022.968687

**Published:** 2022-07-18

**Authors:** Fei Ye, Min Lin, Jia Jin, Sylvain Broussy

**Affiliations:** ^1^ College of Life Sciences and Medicine, Zhejiang Sci-Tech University, Hangzhou, China; ^2^ School of Pharmaceutical Sciences, Zhejiang Chinese Medical University, Hangzhou, China; ^3^ University of Paris, Pharmacy, Paris, France

**Keywords:** computer-aided drug design, lead compound discovery, hit optimization, allosteric regulation, conformational dynamics, artificial intelligence

Owing to the rapid improvement of computational methodologies and high-performance computational resources, computer-aided drug design (CADD) has been validated as an efficient and powerful strategy in almost every stage of drug discovery and development.

Generally, CADD can be divided into structure-based drug design (SBDD) and ligand-based drug design (LBDD). Due to the rapid development of crystallography and homology modeling, structure-based virtual screening has emerged a useful technique to identify potential hits during early stage of drug discovery. LBDD strategies based on available information of known bioactive molecules, such as QSAR (Quantitative Structure–Activity Relationship) analysis, scaffold hopping, pharmacophore modeling, are also widely used for hit optimization and activity prediction. In addition, computational techniques like quantum chemistry calculation, molecular dynamics (MD) simulations and elastic network models can be used to study protein catalytic mechanism, conformational transition and allosteric regulations at an atomic level of detail, which provide useful information for mechanism-based drug design. Recently, with the development of machine learning theory and the accumulation of pharmacological data, artificial intelligence (AI), a powerful data mining technology, has been widely used in various fields of drug design, including virtual screening, *de novo* drug design, QSAR analysis, as well as in silico evaluation of absorption, distribution, metabolism, excretion and toxicity (ADME/T) properties.

In this Research Topic, we have invited some scientists worldwide to contribute original research and review articles which could enhance our understanding of some of the above issues. Several studies utilized multiple computational approaches, such as molecular docking, DFT calculations, molecular dynamics (MD) simulations, ADME/T prediction, as well as biological evaluations to identify novel compounds against a series of important targets, like Tubulin (Khattab and Al-Karmalawy), Dengue Virus NS5 protein (García-Ariza et al.), α-Glucosidase (Liu et al.), hACE2 receptor of SARS-CoV-2 (Al-Karmalawy et al.), Fascin (Lin et al.), TMPRSS2 (Mahmudpour et al.) and Alzheimer’s disease targets (Pradeep et al.). Santana et al. discussed the development of computational approaches to explore the chemo-structural diversity of natural products. CADD methods are also widely used for exploring interactions between ligand and receptor, as well as inhibition mechanisms of active compounds. Tao et al. performed molecular docking and MD simulations to study the interaction between RBD and two glycopeptide antibiotics (Vancomycin and Teicoplanin). Wang et al. used network pharmacology and molecular docking to explore the mechanism of Shan Ci Gu (Cremastra appendiculata) against non-small cell lung cancer. In addition, Di Filippo et al. proposed a machine learning model to predict drug transfer across the human placenta barrier, which could be used as a filter for chemical libraries in virtual screening campaigns.

In summary, the above works presented in this special Research Topic illustrate the applications of CADD approaches and highlight the importance of developing new methods. At last, as the Guest Editors of this Research Topic, we would like to thank all the authors for their contributed articles and all the referees for their comments on the manuscripts. We hope that the readers will find this Research Topic interesting and useful for their research. Finally, we appreciate the editorial staff of Frontiers in Chemistry for their work in publishing this Research Topic.

